# ICTV Virus Taxonomy Profile: *Hepeviridae*

**DOI:** 10.1099/jgv.0.000940

**Published:** 2017-10-12

**Authors:** Michael A. Purdy, Tim J. Harrison, S. Jameel, X-J. Meng, H. Okamoto, W. H. M. Van der Poel, Donald B. Smith, ICTV Report Consortium

**Affiliations:** ^1^​ Division of Viral Hepatitis, Centers for Disease Control and Prevention, MS-A33, 1600 Clifton Rd NE, Atlanta, GA 30329, USA; ^2^​ University College London, Gower St, Bloomsbury, London, WC1E 6BT, UK; ^3^​ Wellcome Trust/DBT India Alliance, 1110 DLF Tower B, Jasola, New Delhi 110025, India; ^4^​ College of Veterinary Medicine, Virginia Polytechnic Institute and State University, Blacksburg, VA, USA; ^5^​ Division of Virology, Department of Infection and Immunity, Jichi Medical University School of Medicine, Tochigi-ken, Japan; ^6^​ Wageningen Bioveterinary Research, Wageningen University and Research, Lelystad, The Netherlands; ^7^​ Ashworth Laboratories, University of Edinburgh, Charlotte Auerbach Road, Edinburgh, EH9 3FL, Scotland, UK

**Keywords:** *Hepeviridae*, ICTV, taxonomy, hepatitis E virus, swine hepatitis E virus, avian hepatitis E virus, piscihepevirus

## Abstract

The family *Hepeviridae* includes enterically transmitted small non-enveloped positive-sense RNA viruses. It includes the genera *Piscihepevirus*, whose members infect fish, and *Orthohepevirus*, whose members infect mammals and birds. Members of the genus *Orthohepevirus* include hepatitis E virus, which is responsible for self-limiting acute hepatitis in humans and several mammalian species; the infection may become chronic in immunocompromised individuals. Extrahepatic manifestations of Guillain–Barré syndrome, neuralgic amyotrophy, glomerulonephritis and pancreatitis have been described in humans. Avian hepatitis E virus causes hepatitis–splenomegaly syndrome in chickens. This is a summary of the International Committee on Taxonomy of Viruses (ICTV) Report on the taxonomy of the *Hepeviridae*, which is available at www.ictv.global/report/hepeviridae.

## Virion

**Fig. 1.**
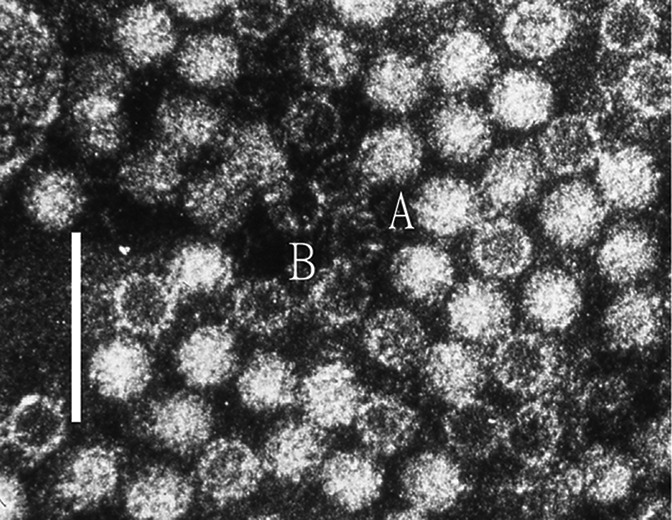
Negative contrast electron micrograph of human hepatitis E virus virions from a case stool collected in Nepal. (A) virion and (B) empty capsid. The bar represents 100 nm (photograph from M. Purdy).

The virions of human hepatitis E virus are icosahedral, non-enveloped, spherical particles with a diameter of approximately 27–34 nm (Table 1, Fig. 1). The capsid is formed by capsomeres consisting of homodimers of a single capsid protein, forming the virus shell. Each capsid protein contains three linear domains forming distinct structural elements: S (the continuous capsid), P1 (three-fold protrusions) and P2 (two-fold spikes). Neutralizing epitopes have been found in the P2 domain. Each domain contains a putative polysaccharide-binding site that may interact with cellular receptors. Native *T*=3 capsids contain flat dimers, with less curvature than those of *T*=1 virus-like particles [1].

**Table 1.** Characteristics of the family *Hepeviridae*

Typical member:	human hepatitis E virus Burma (M73218), species *Orthohepevirus A,* genus *Orthohepevirus*
Virion	Non-enveloped, 27–34 nm diameter with a single capsid protein
Genome	6.4–7.2 kb capped positive-sense monopartite RNA containing three ORFs
Replication	Occurs in association with the host endoplasmic reticulum
Translation	From genomic (ORF1) and a subgenomic (ORF2 and ORF3) capped mRNA
Host range	Mammals (*Orthohepevirus A*, *C* and *D*), birds (*Orthohepevirus B*) and trout (*Piscihepevirus*)
Taxonomy	Two genera

## Genome

**Fig. 2.**
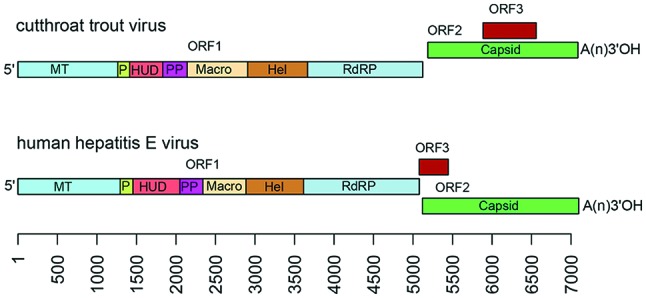
Genome organization of cutthroat trout virus and human hepatitis E virus. A short 5′ non-coding region is followed by ORF1, encoding non-structural proteins including the putative functional domains: MT, methytransferase; P, a putative papain-like cysteine protease; HUD, *Hepeviridae* unique domain, also called the Z domain [7]; PP, a hypervariable polyproline region that is dispensable for virus infectivity; Macro, macro domain; Hel, helicase; and RdRP, RNA-dependent RNA polymerase [7, 8]. ORF2 encodes a capsid protein and is followed by a short 3′ NCR. ORF3 overlaps ORF2 in a different reading frame and encodes a small phosphoprotein with a multi-functional C-terminal region. The scale is in bases.

Viral genomes are positive-sense monopartite RNA of about 6.4 to 7.2 kb, with three ORFs flanked by short 5′- and 3′-terminal non-coding regions: ORF2 overlaps ORF3 but neither overlaps ORF1. The 5′-end is m^7^G-capped and the 3′-end is polyadenylated (Fig. 2). Non-structural proteins encoded by the first ORF (ORF1) have limited similarity with the ‘alpha-like supergroup’ of viruses and contain domains consistent with a methyltransferase, papain-like cysteine protease, macro domain, RNA helicase and RNA-dependent RNA polymerase [2]. Some of these properties have been confirmed experimentally. It remains unclear whether the ORF1-encoded activities function as a single protein with multiple functional domains or as individually cleaved smaller proteins. Virions are constructed from a capsid protein encoded by ORF2 that may be proteolytically processed. A small immunoreactive protein (12.5 kDa) encoded by the third ORF (ORF3) has been shown to exhibit multiple functions associated with virion morphogenesis, egress and viral pathogenesis. The capsid and ORF3 proteins are translated from a subgenomic RNA that is generated from the genome. Although human hepatitis E viruses are shed into faeces as non-enveloped virions, they appear to be released into the bloodstream as membrane-associated virions [3].

## Replication

The replication of human hepatitis E virus is not well understood. The viral RNA-dependent RNA polymerase associates with the host endoplasmic reticulum through residues encoding a predicted transmembrane domain in order to begin replicating the viral genome. It appears that replication involves temporal separation and alternating cycles of positive- and negative-sense RNAs to produce capsid, ORF3 protein, ORF1 polypeptide and new genomes, resulting in the generation of progeny virions [2, 4].

## Taxonomy

*Orthohepevirus*. Members of this genus infect a wide range of mammals, including humans, domestic and wild swine, deer, sheep, rabbits, camels, mongooses, (*Orthohepevirus A* members), rats, ferrets, shrews, bandicoots, mink (*Orthohepevirus C* members), bats (*Orthohepevirus D* members), and birds (*Orthohepevirus B* members) [5]. Unclassified viruses have been detected in moose and foxes, and in droppings from little egrets and kestrels. Human hepatitis E virus can cause self-limiting acute hepatitis in humans and is transmitted by contaminated water or the consumption of undercooked or raw meat and dairy and other products from infected animals. Human hepatitis E virus is the leading cause of acute hepatitis in developing countries [6].

*Piscihepevirus*. This genus includes a single species whose typical isolate, cutthroat trout virus, infects trout, although its pathogenicity and full host range are unknown [5].

## Resources

Full ICTV Online (10th) Report: www.ictv.global/report/hepeviridae.
